# Crystal Structure of DIM-1, an Acquired Subclass B1 Metallo-β-Lactamase from *Pseudomonas stutzeri*

**DOI:** 10.1371/journal.pone.0140059

**Published:** 2015-10-09

**Authors:** Michael P. S. Booth, Magda Kosmopoulou, Laurent Poirel, Patrice Nordmann, James Spencer

**Affiliations:** 1 School of Cellular and Molecular Medicine, University of Bristol Medical Sciences Building, University Walk, Bristol, BS8 1TD, United Kingdom; 2 Medical and Molecular Microbiology Unit, Department of Medicine, Faculty of Science, University of Fribourg, Rue Albert Gockel 3, CH-1700, Fribourg, Switzerland; Monash University, AUSTRALIA

## Abstract

Metallo-β-lactamases (MBLs) hydrolyze almost all classes of β-lactam antibiotic, including carbapenems—currently first choice drugs for opportunistic infections by Gram-negative bacterial pathogens. MBL inhibitor development is complicated by the diversity within this group of enzymes, and by the appearance of new enzymes that continue to be identified both as chromosomal genes and on mobile genetic elements. One such newly discovered MBL is DIM-1, a mobile enzyme originally discovered in the opportunist pathogen *Pseudomonas stutzeri* but subsequently identified in other species and locations. DIM-1 is a subclass B1 MBL more closely related to the TMB-1, GIM-1 and IMP enzymes than to other clinically encountered MBLs such as VIM and NDM; and possesses Arg, rather than the more usual Lys, at position 224 in the putative substrate binding site. Here we report the crystallization and structure determination of DIM-1. DIM-1 possesses a binuclear metal center with a 5 (rather than the more usual 4) co-ordinate tri-histidine (Zn1) site and both 4- and 5-co-ordinate Cys-His-Asp- (Zn2) sites observed in the two molecules of the crystallographic asymmetric unit. These data indicate a degree of variability in metal co-ordination geometry in the DIM-1 active site, as well as facilitating inclusion of DIM-1 in structure-based MBL inhibitor discovery programmes.

## Introduction

Acquired metallo-β-lactamases (MBLs) present a global public health challenge [1]. Their high hydrolytic activity against carbapenems, (broad-spectrum β-lactams of ultimate choice for Gram-negative infections), and their non-susceptibility to serine β-lactamase inhibitors, make their dissemination significant in the rise of antibiotic resistant pathogens. Sequence data define three MBL subclasses (B1 –B3 [2]) with B1 including almost all enzymes whose genes are found on mobile genetic elements, and thus capable of rapid dissemination across geographic and species boundaries. B1 MBLs from the IMP, VIM and NDM groups have already been found in multiple hosts, both Enterobacteriaceae [3] and non-fermenters (*Pseudomonas aeruginosa*, *Acinetobacter baumannii* [4–6]), while additional mobile enzymes [7–9] continue to be identified.

**Fig 1:**
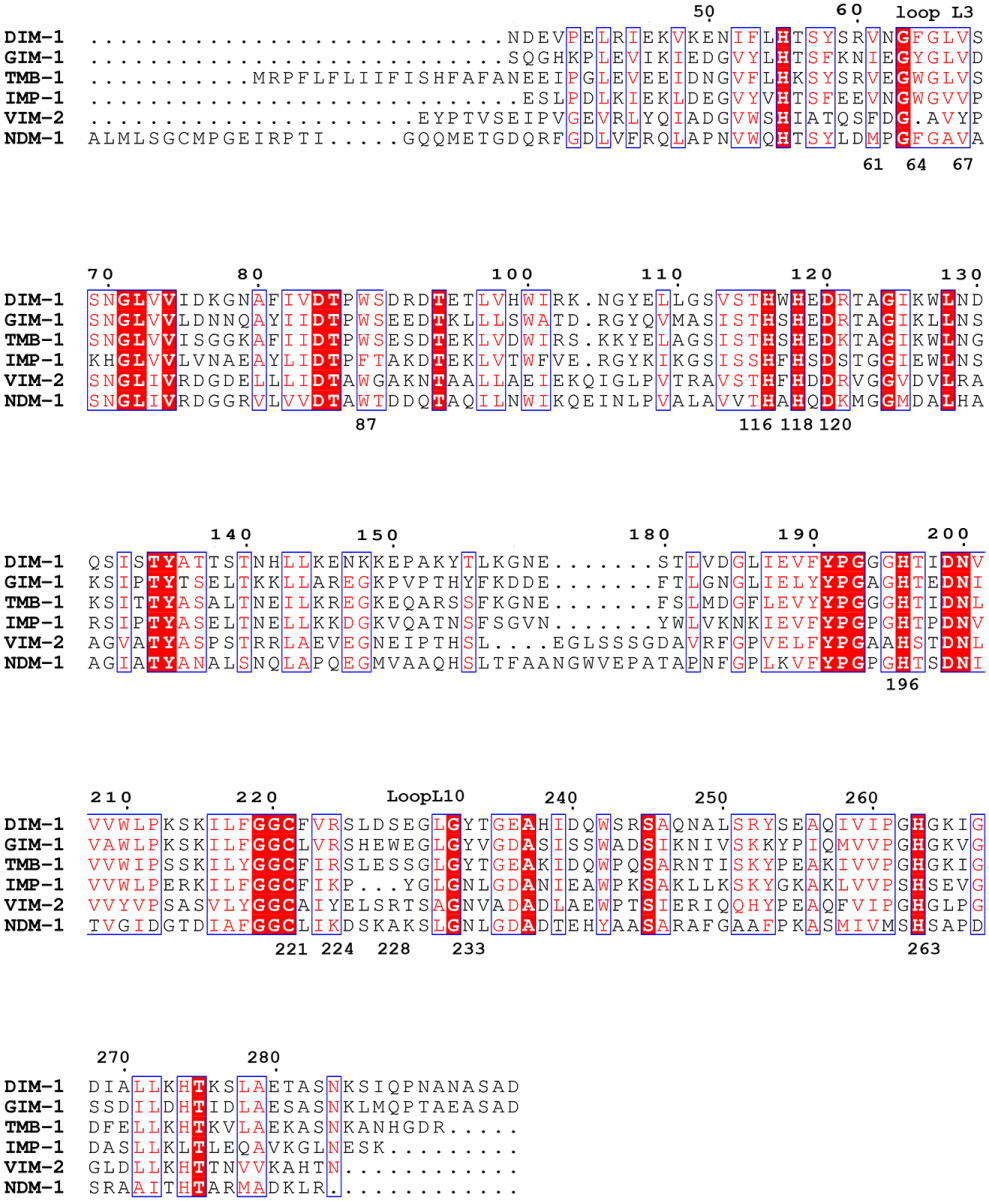
Alignment of DIM-1 with Selected Subclass B1 Metallo-β-Lactamases. Invariant residues are highlighted on a red background; conservative substitutions are colored red. Residue numbers according to the Class B β-Lactamase (BBL) standard numbering scheme are positioned above the sequences; individual residues discussed in the text are numbered below the sequences. This Figure was prepared using ESPript [15].

DIM-1 (Dutch IMipenemase) was identified in a carbapenem non-susceptible isolate of the occasional opportunist pathogen *Pseudomonas stutzeri*, recovered from a patient undergoing surgery for tibial osteomyelitis [10]. The carbapenemase gene, *bla*_*DIM-1*_ (GenBank: GU323019.1), resided on a class 1 integron on a 70 kb plasmid, suggesting a capability for dissemination, and has subsequently been identified in isolates of multiple species (*Escherichia coli*, *Enterobacter cloacae*, *Comomonas testosteroni*) of African origin and in a single strain of *P*. *stutzeri* from India [11, 12]. Independent acquisition events from a currently unknown environmental reservoir have been suggested to account for these apparently unconnected identifications in widely separated locations [11]. The *bla*_*DIM-1*_ gene encodes a subclass B1 MBL resembling TMB-1 (67% identity [7]) GIM-1 (54% identity [13]) and IMP-1 (48% identity [14]; percentage identities are for the mature, processed polypeptides), but more distantly related to VIM-1 and NDM-1. An alignment of selected B1 MBLs is presented in Fig 1.

Like other B1 MBLs, DIM-1 is a broad-spectrum enzyme, hydrolyzing penicillins, cephalosporins and carbapenems, although compared to some enzymes turnover of some penicillins is reduced and activity is weaker against cephalosporins, e.g. ceftazidime or cefepime, with bulky, charged, 3’ substituents [10]. In common with GIM-1 and TMB-1, DIM-1 is distinguished from other B1 enzymes by possession of arginine at position 224, rather than the lysine (Lys224) found in enzymes such as the IMP and NDM groups. Recent crystal structures [16, 17] show NDM-1 Lys224 to make electrostatic interactions with the C3 carboxylate of hydrolyzed carbapenems, suggesting that this substitution might affect substrate binding. Here we report the crystal structure of DIM-1, facilitating its inclusion in structure-based inhibitor discovery programmes.

## Materials and Methods

### Protein Expression

DIM-1 was purified from *E*. *coli* TOP10 containing the plasmid pXD-1 [10] by ion-exchange and size exclusion chromatography in a modification of previously published procedures. Briefly, 2 L of Luria-Bertani broth were inoculated with 10 mL / L of an overnight culture and grown for 24 h at 37°C with shaking at 160 rpm. Cells were harvested by centrifugation (4 000 g, 20 min, 4°C), washed in phosphate-buffered saline (PBS) and frozen until required. Thawed pellets were resuspended in 25 mL / L Buffer A (50 mM Tris-Cl pH 7.5) containing protease inhibitors (Complete EDTA-free, Roche Life Science, Burgess Hill, U.K.) and lysed by sonication. The lysate was clarified by centrifugation (15,000 g, 30 min, 4°C) and dialyzed overnight (4°C) against 1 L buffer A. The dialysate was loaded onto a 50 mL Q-sepharose column (Sigma, Poole, U.K.) which was washed in buffer A until absorbance returned to baseline. Bound protein was eluted in buffer A using a 300 mL 0–0.5 M NaCl gradient; DIM-1 eluted at approximately 0.1 M NaCl as identified by hydrolysis of the chromogenic cephalosporin nitrocefin [18]. DIM-1 containing fractions were concentrated by centrifugal ultrafiltration, loaded onto a 300 mL Superdex-75 column pre-equilibrated in Buffer B (50 mM Tris-Cl 200 mM NaCl pH 7.5) and eluted in the same buffer. The purity of the final preparation was greater than 95% as adjudged by SDS—PAGE [19]. The purified protein was concentrated for crystallization trials by centrifugal ultrafiltration.

### Crystallization and Structure Solution

Sparse matrix crystallization screening with the Morpheus suite [20] used a Pheonix robot (Art-Robbins) to set sitting drops (0.5 μL each of DIM-1 (15 mg / mL) and reservoir solution) in 96-well plates with 100 μL well volume. Crystals appeared after 5–10 days’ incubation (20°C) in 20% polyethylene glycol (PEG) 550 monomethyl ether (MME) / 10% PEG 20 000; 50 mM HEPES / 50 mM MOPS pH 7.5; were mounted in rayon loops, snap-frozen and stored in liquid nitrogen until required. For soaking experiments, ceftazidime (Sigma, Poole, U.K.) dissolved to 250 mM in dimethyl sulfoxide (DMSO) was added to the drop to a final concentration of 85 mM and 33% DMSO and crystals frozen after 1 hour incubation at 20°C. Diffraction data were collected on an ADSC Quantum 4 detector mounted on beamline I03 of Diamond Light Source (Didcot, U.K.) and reflections integrated, scaled and merged using HKL2000 [21]. Phases were obtained by molecular replacement using PHASER [22] with IMP-1 (PDB accession 1DDK; [23]) as a search model. The model was built using Coot [24] and refined using Refmac5 [25] as implemented in the CCP4 software suite [26] with TLS (Translation/Libration/Screw) groups identified by the TLSMD server [27, 28]. The quality of the final model was assessed using PROCHECK [29] and Molprobity [30]. Data collection and refinement statistics are given in Table 1. Co-ordinates and structure factors have been deposited with the Protein Data Bak (www.rcsb.org) with accession codes 4WD6 (native structure) and 4ZEJ (ceftazidime-exposed structure).

**Table 1:** Data Collection and Refinement Statistics.

Dataset	Dataset 1	Dataset 2
Processing	HKL2000 [21]	HKL2000
Beamline	DLS I03	DLS I03
Space Group	P 2_1_ 2_1_ 2_1_	P 2_1_ 2_1_ 2_1_
Cell Dimensions (Å)	a = 47.72 b = 47.86 c = 185.22	a = 46.40 b = 46.98 c = 183.76
Wavelength (Å)	0.9763	0.9763
Resolution (Å)	50.00–2.20 (2.28–2.20)^a^	50.00–1.80 (1.86–1.80)^a^
Total reflections	149 531	496 348
Unique reflections	21 133 (1 504)^a^	38 481 (3 667)^a^
Completeness (%)	94.5 (68.4)^a^	99.6 (96.6)^a^
Redundancy	7.1 (5.9)^a^	12.9 (7.0)^a^
I / (sig. I)	32.5 (6.6)^a^	29.6 (2.5)^a^
R_merge_ (%)	5.1 (25.1)^a^	8.8 (50.1)^a^
Refinement	REFMAC5	REFMAC5
Total reflections	20 006 (1 016)^a^	36 522 (2 499)^a^
Resolution (Å)	42.55–2.20 (2.26–2.20)^a^	91.88–1.79 (1.84–1.79)^a^
R_cryst_ (%)	20.7 (23.9)^a^	17.2 (21.7)^a^
R_free_ (%)^b^	27.4 (34.2)^a^	18.9 (22.8)^a^
RMS bond length (Å)	0.0138	0.0078
RMS bond angle (Å)	1.6167	1.2289
Protein atoms	3 392	3 434
Water molecules	126	192
% residues in Ramachandran regions (favored/allowed/disallowed)	95.3/4.2/0.5	97.2/2.3/0.5
B-factors (protein)^c^	47.38 (55.26)	30.98 (39.81)
B-factors (water molecules)	48.57	38.68
PDB accession code	4WD6	4ZEJ

^a^ Data for the highest resolution shell are in parentheses.

^b^ R_free_ was calculated with 5% of the reflections omitted.

^c^Figures in parentheses are for chain B.

## Results and Discussion

DIM-1 crystallized in space group P 2_1_ 2_1_ 2_1_ with crystals containing two molecules in the asymmetric unit (Table 1). The structure was solved by molecular replacement and could be built as two polypeptide chains. Two complete data sets were collected, a native data set from a crystal diffracting to 2.2 Å resolution (dataset 1) and a second, higher resolution data set (dataset 2) arising from one of a number of crystals that had been soaked with the cephalosporin ceftazidime in DMSO, but for which no electron density corresponding to a bound β-lactam could be resolved. As this crystal diffracted to higher resolution (1.8 Å) and yielded higher quality data (Table 1) this structure was also solved and refined. Non-crystallographic symmetry was not employed at any stage in the refinement process; therefore our data provide structures for four independently refined DIM-1 monomers. The final models contain 219 (residues 39–294 according to the standard class B β-lactamase (BBL) numbering scheme [2]) amino acids per chain, apart from chain B of the lower resolution structure (dataset 1) for which weak electron density meant that residues 25–28 (60–64; BBL residue numberings are given in parentheses throughout this paper) inclusive were not modelled. Overall Cα rms deviations between the four chains were a maximum 0.36 Å. For the four chains (A and B of dataset 1 and A and B of dataset 2, respectively) 95/ 94/ 97/ 97% of residues lie within the most favored regions of the Ramachandran plot and a further 4/ 6/ 2/ 2% within the additionally allowed regions [30]. In three of the four chains Asp-48 (84) lies in the disfavored Ramachandran region due to its involvement in a buried hydrogen bond network, involving Ser-33 (69), Asn-34 (70), Thr-76 (115), Arg-82 (121) and Gly-199 (262) that is common to most members of the B1 MBL subfamily and connects the two halves of the protein beneath the active site [31].

**Fig 2:**
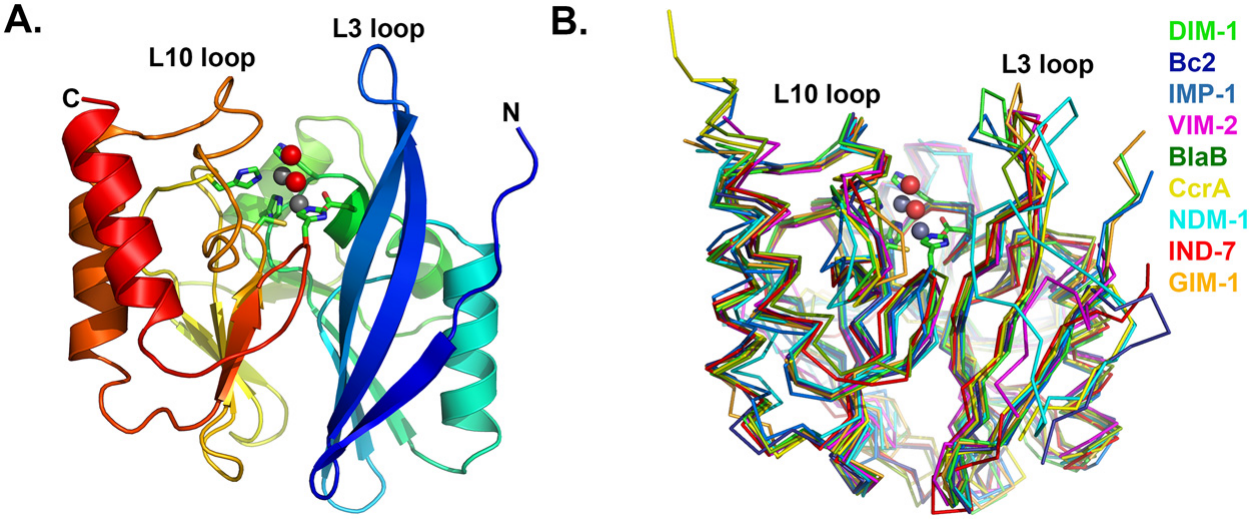
Crystal Structure of DIM-1. A. Overall fold of the enzyme. Protein backbone is color-ramped from blue (N-) to red (C-terminus). Active site residues are rendered as sticks (carbon atoms in green, other atom colors as standard). Zinc ions (gray) and water molecules (red) are shown as spheres. B. Superposition of B1 MBL structures: DIM-1 (green); BcII (pdb accession 1BC2 ([43], blue); IMP-1 (1DDK [23], teal); VIM-2 (1KO3 [44], magenta); BlaB (1M2X [45], dark green); CcrA (1ZNB [46], yellow); NDM-1 (3Q6X [41], cyan); IND-7 (3L6N [47], red) and GIM-1 (2YNT [48], orange). This Figure was generated using Pymol (www.pymol.org).

DIM-1 adopts the αβ/βα sandwich fold of the MBL hydrolase superfamily, with the active site situated in the groove between the two domains and containing two zinc ions (Fig 2A). Superposition (using SSMsuperpose [32]) with other available B1 MBL structures (Fig 2B) indicates that DIM-1 most closely resembles GIM-1 (Cα rmsd 0.87 Å) and IMP-1 (Cα rmsd 0.91 Å). Among B1 enzymes the MBL fold is well conserved, with significant differences apparent at the N-terminus, in the length of the C-terminal α-helix, and in the flexible loop L3 (residues 25–32 (60–68)) that connects the β2 and β3 strands. Crystallographic [23, 33–35], mutagenic and kinetic [36, 37], NMR [38, 39] and molecular dynamics [40] evidence indicates that this loop adjusts its conformation on β-lactam or inhibitor binding and contributes to substrate affinity and specificity. In the lower resolution (dataset 1) DIM-1 structure the L3 loop could not be modelled for chain B, but in chain A it extends over the active site groove in a “closed” conformation more typical of MBL complexes [17, 34, 35, 41] than of other uncomplexed B1 MBL structures. These differences may arise from differences in crystal contacts- while the apex of loop L3 of chain A lies close both to chain B and symmetry related molecules, fewer contacts are made by the equivalent subunit of chain B. In the higher resolution structure obtained from dataset 2, the complete loop L3 could be modeled for both chains, but chain B featured elevated B-factors consistent with a reduced involvement in crystal contacts. Establishing whether the observed conformation of the DIM-1 L3 loop arises primarily from crystal contacts, or indeed reflects that adopted on substrate and/or inhibitor binding, must await determination of structures for the relevant complexes.

**Fig 3:**
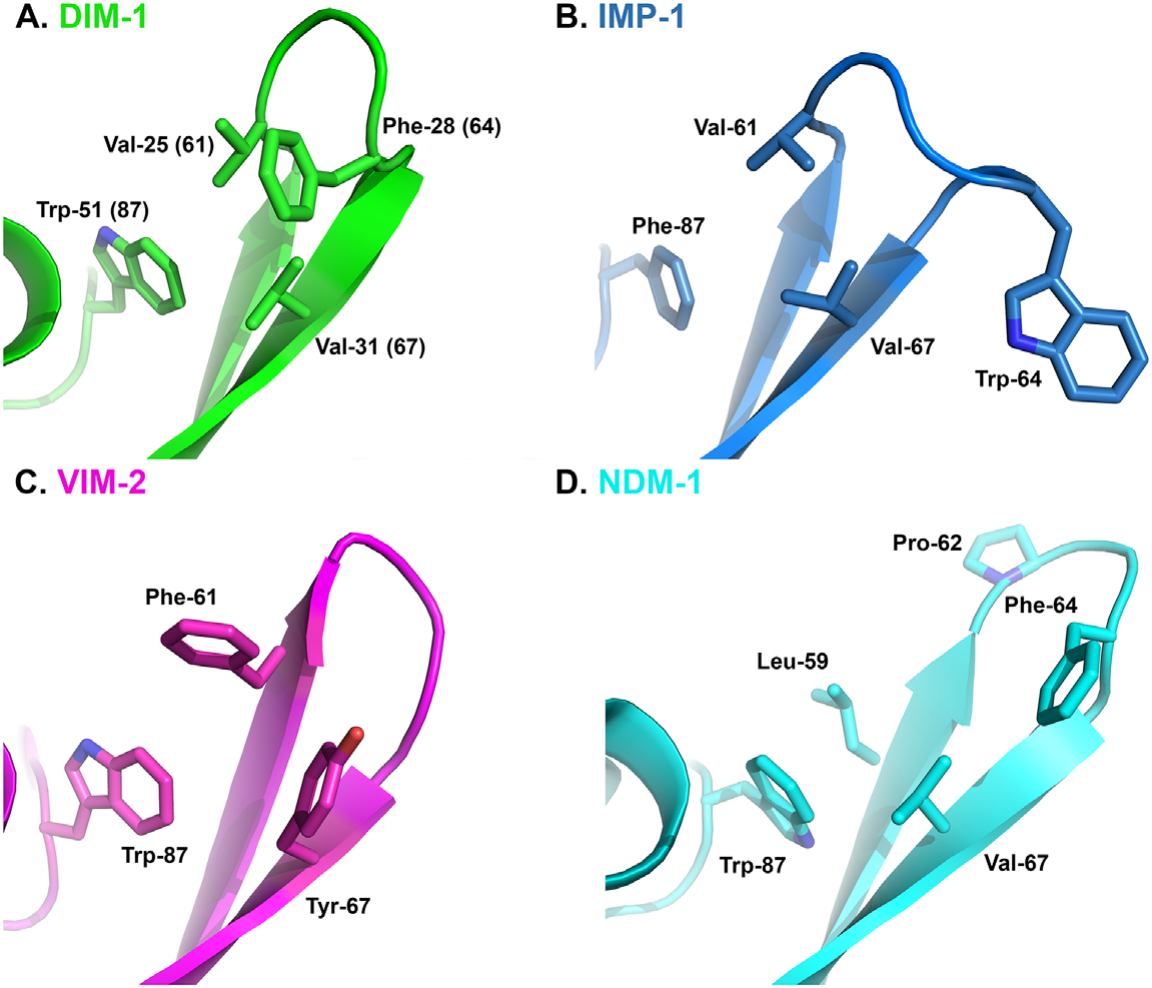
L3 Loop Conformations in Subclass B1 Metallo-β-Lactamases. A. DIM-1 (green); B. IMP-1 (1DDK [23], teal); C. VIM-2 (1KO3 [44], magenta); D. NDM-1 (3Q6X [41], cyan). This Figure was generated using Pymol.

**Fig 4:**
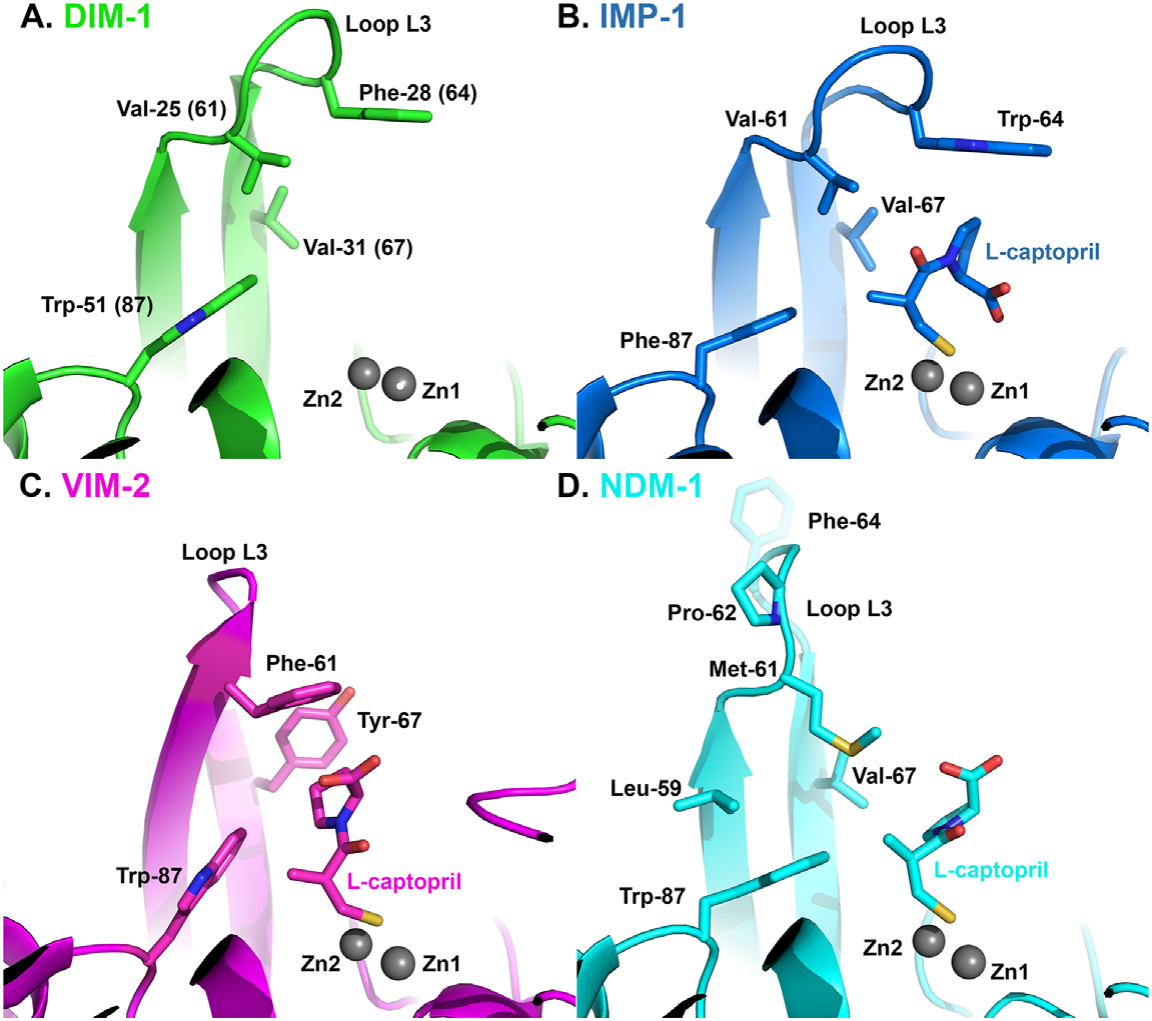
Comparison of DIM-1 Active Site with L-Captopril-bound Complexes of Subclass B1 Metallo-β-Lactamases. A. DIM-1 (green); B. IMP-1 (4C1F, teal); C. VIM-2 (4C1D, magenta); D. NDM-1 (4EXS [17], cyan). This Figure was generated using Pymol.

Our structure also confirms that DIM-1 features hydrophobic aliphatic amino acids (valine) at positions 25 (61) and 31 (67) at the base of the L3 loop, and an aromatic residue (phenylalanine) at position 28 (64) at its apex. In this DIM-1 resembles IMP-1 (and similar enzymes such as GIM-1 [13] or KHM-1 [9]) rather than the VIM enzymes (where positions 25 (61) and 31 (67) are occupied by aromatic residues) or NDM-1 (where the L3 loop contains proline at position 26 (62) and adopts a different conformation; Fig 3). As discussed above, inhibitor binding to enzymes such as CcrA [33] or BcII [39] can involve conformational changes in the L3 loop, and be affected by mutations within it [36]. Moreover, comparisons of inhibition of multiple B1 enzymes that differ in their L3 loops (including NDM, IMP and VIM, but not DIM, enzymes) by a series of hydroxythiazole compounds identified differences between enzymes of up to two orders of magnitude in IC_50_ values [42]. Unfortunately, limited structural data are available that permit direct comparison of inhibitor binding to different MBL targets. However, structures for complexes of the thiol L-captopril with IMP-1 (pdb id 4C1F), NDM-1 (pdb 4EXS [17]) and VIM-2 (pdb 4C1D) show that this adopts different conformations that are in part imposed by the proximity of different residues on the L3 loop (residues Met-61 of NDM-1; Val-61 of IMP-1 and Phe-62 and Tyr-67 of VIM-2) to the captopril pyrrolidine ring. Comparison of these structures with that of DIM-1 (Fig 4) suggests that positioning of Phe-28(64) on loop L3 over the active site, together with the presence of Val-25 (61), Val-31 (67) and Trp-51(87) would support an orientation for the binding of the captopril pyrrolidine to DIM-1 that is similar to that adopted upon binding to IMP-1, and that differs from those observed with VIM-2 or NDM-1. It will be of interest to establish the extent to which this similarity between the L3 loops of IMP-1 and DIM-1 results in similar inhibition profiles, and similar modes of inhibitor binding.

**Fig 5:**
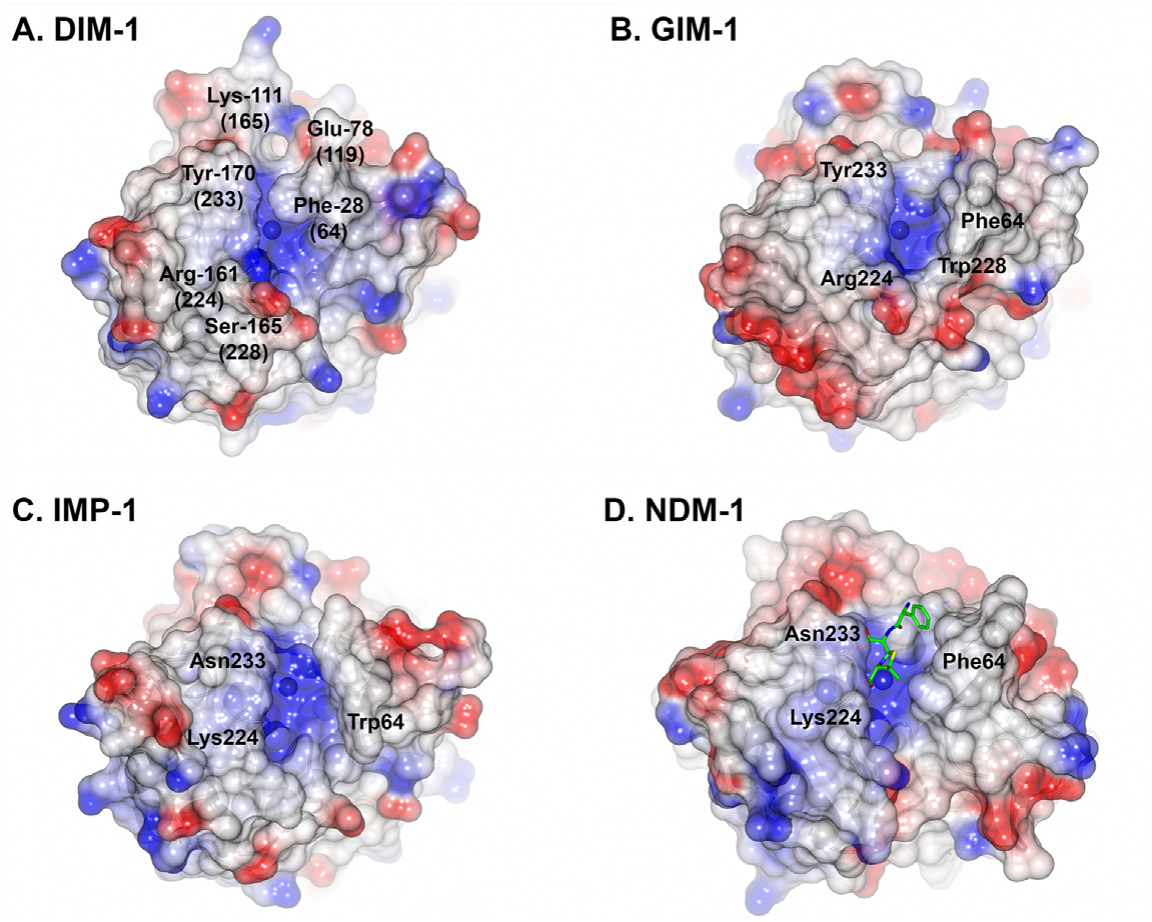
Active Site Grooves of B1 Metallo-β-Lactamases. Figure shows surfaces of A. DIM-1; B. GIM-1 (pdb accession 2YNT [48]) C. IMP-1 (1DDK [23]) and D. NDM-1 (3Q6X [41]) colored according to electrostatic potential from red (- 0.5 V) to blue (+ 0.5 V). Hydrolyzed ampicillin bound to NDM-1 is shown as sticks (carbon atoms green, other atom colors as standard). This Figure was generated using CCP4MG [50].

Loop L10 (residues 160–176 (223–239)) on the opposite side of the active site groove also shows features that distinguish DIM-1 from related enzymes. The DIM-1 L10 loop lacks Trp-165 (228), which in GIM-1 restricts this part of the active site cavity [48] and instead has serine at this position. Like GIM-1, DIM-1 possesses tyrosine, rather than the more usual asparagine, at position 170 (233), which further constrains the active site groove and provides a hydrophobic surface for substrate interactions, but abolishes the possible electrostatic contacts between substrate and the Asn-170 (233) side chain that have been proposed for some other B1 enzymes ([17, 35, 49]. The DIM-1 active site also features a close electrostatic contact between the side chains of Glu-78 (119) and Lys-111 (165), forming a bridge across the active site groove in the vicinity of the probable binding site for substituent groups at the R1 position of the β-lactam core (i.e. the C6/C7 substituents of penicillins or cephalosporins, respectively). Taken together, these factors create an active site in DIM-1 that is narrower and more defined than the relatively wide open groove of many other B1 MBLs (Fig 5).

**Fig 6:**
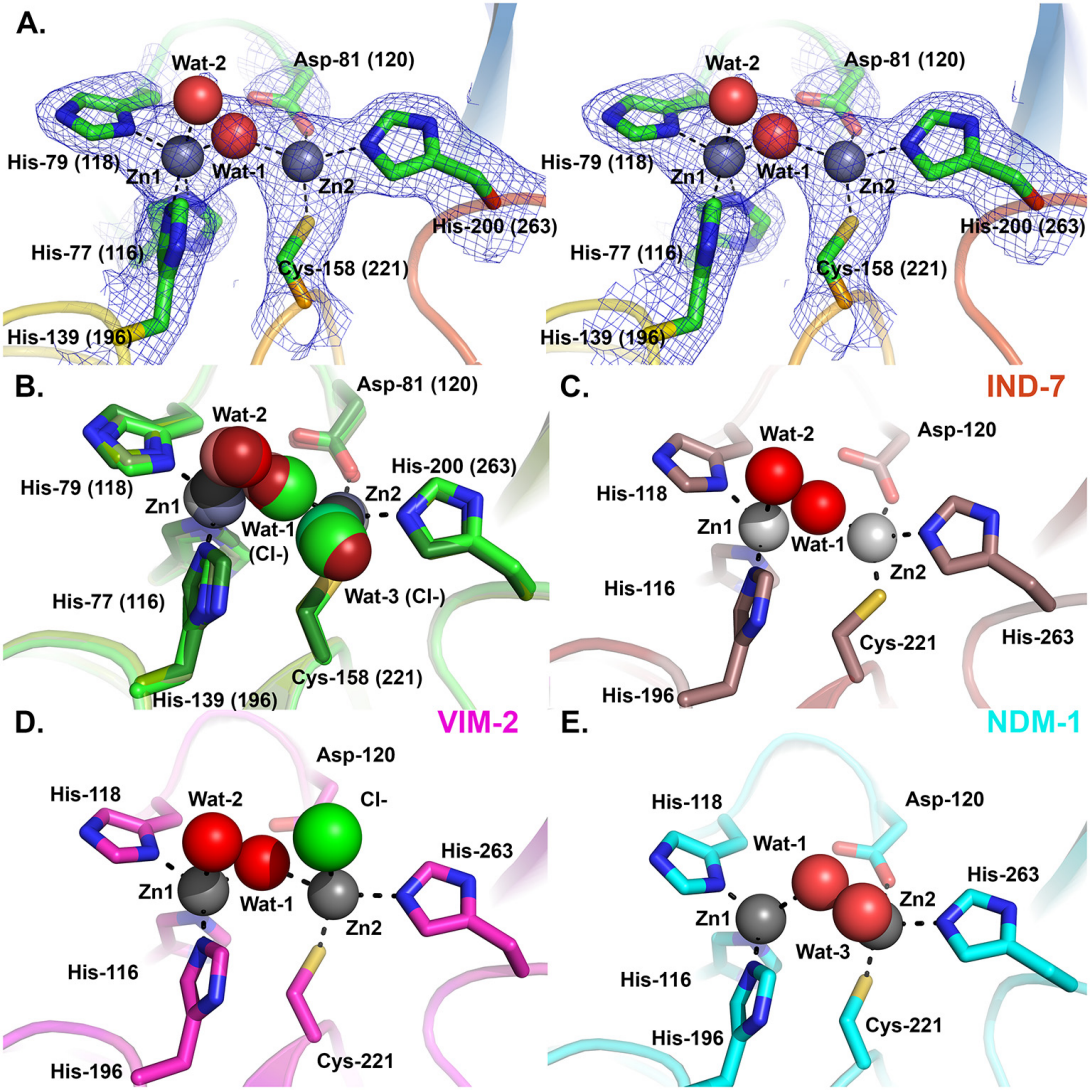
DIM-1 Active Site and Comparison with Selected B1 Metallo-β-Lactamases. A. Stereogram of DIM-1 active site (dataset 1 structure). Carbon atoms are colored green, zinc ions gray, water molecules red, other atom colors as standard. Electron density map shown is 2|Fo|-|Fc|.ϕ_calc_, contoured at 1.0 σ. B. Overlay of DIM-1 active sites for the four independently refined molecules. Structures from dataset 1 are in darker, and dataset 2 in lighter, shades. Dashed lines show metal:ligand interactions in chain A of dataset 1. Note presence of additional water molecule (Wat-3; dataset 1 chain B) or chloride ions (dataset 2 structures) bound to Zn2 ion, and bridging chloride ion in dataset 2 chain A structure. C. Active site of IND-7 (3L6N [47]). Note five- and four-fold co-ordination of Zn1 and Zn2, respectively. D. Active site of VIM-2 (1KO3 [44]). Not five-fold co-ordination of both Zn^2+^ ions. E. Active site of NDM-1 (3PSU [54]) Note four- and five-fold co-ordination of Zn1 and Zn2, respectively. This Figure was generated using Pymol.

Electron density maps (Fig 6A) unequivocally identify two metal ions in the DIM-1 active site, consistent with the binuclear species being the physiological form of B1 MBLs [51, 52]. As no particular precautions were taken to avoid oxidation of the ligand Cys-158 (221), which is associated with loss of the metal ion in the Zn2 site in certain B1 MBLs, we surmise that this occurs less readily in DIM-1 than in some other enzymes such as BcII, SPM-1 or VIM-2 [31, 45]. All active site metal ions were refined as zinc, with occupancies of 1.0. This procedure yielded B-factors for the individual zinc ions of 42.58 and 48.04 Å^2^ (chain A Zn1 and Zn2 respectively, dataset 1); 50.73 and 57.25 Å^2^ (chain B Zn1 and Zn2 respectively, dataset 1); 23.43 and 26.41 Å^2^ (chain A Zn1 and Zn2 respectively, dataset 2); and 27.13 and 36.58 Å^2^ (chain B Zn1 and Zn2 respectively, dataset 2). This compares with B-factors for the complete individual protein chains of 47.38 and 55.26 Å^2^ (chains A and B; dataset 1) and 30.98 and 39.81 Å^2^ (chains A and B; dataset 2). The consistency between the B-values obtained for the modelled zinc ions and the respective protein chains suggests that refining the bound metal ions as zinc is reasonable. However, while we consider zinc to be the most likely metal ion to be present in the DIM-1 active site, the available data cannot preclude the possibility that some fraction of other metallated species may be present.

In the four independently refined DIM-1 chains the two metal ions lie approximately 4.00 Å–4.40 Å apart (distances in Table 2) and are liganded by the conserved residues His-77 (116), His-79 (118) and His-139 (196) (tri-histidine or Zn1 site); and Asp-81 (120), Cys-158 (221) and His-200 (263); Cys-His-Asp or Zn2 site). A “bridging” water molecule (or hydroxide ion) that is generally proposed to be the nucleophile in the hydrolytic reaction [53], is positioned between the two metal ions (Fig 6A, Wat1), lying closer to Zn1 that Zn2. (Zinc:ligand distances are given in Table 2). (In chain A of the higher resolution structure (dataset 2) this “bridging” ligand was modelled as a chloride ion (chloride was present in the buffer used for size exclusion chromatography)). These features are largely typical of available crystal structures for binuclear B1 MBLs.

**Table 2:** Metal:ligand distances (Å).

Structure	Dataset 1	Dataset 2
Zn1:His77(116)	2.35^a^ (2.19)^b^	2.16^a^ (2.18)^b^
Zn1:His79(118)	2.05 (2.34)	2.05 (2.16)
Zn1:His139(196)	2.12 (2.32)	2.09 (2.07)
Zn1:Wat1	1.81 (1.89)	2.26^c^ (1.99)
Zn1:Wat2	2.53 (2.40)	2.52
Zn2:Asp81(120)	2.33 (2.60)	2.03 (2.09)
Zn2:Cys168(221)	2.16 (2.11)	2.19 (2.13)
Zn2:His200(263)	2.24 (2.12)	2.15 (2.20)
Zn2:Wat1	2.44 (2.37)	2.27^c^ (2.75)
Zn2:Wat3	(2.43)	2.47^c^ (2.87)^c^
Zn1:Zn2	3.97 (4.02)	4.22 (4.39)
Wat-1:Asp81(120)	2.94 (3.24)	2.98 (3.04)

^a^Chain A.

^b^Figures in parentheses are for chain B.

^c^Refined as a chloride ion

B1 MBLs of known structure show a variety of co-ordination geometries at both the Zn1 and Zn2 sites. In the majority of structures the Zn1 site is tetrahedral [46, 48, 54]. However, in three of the four refined active sites DIM-1 contains a pentaco-ordinate Zn1 center with a second water molecule (Fig 6A, Wat2) acting as an additional ligand but at a distance (~2.4–2.5 Å from Zn1) and with elevated B-factors (where both Wat1 and Wat2 are present (dataset 1) Wat2 B-factors 44.31 Å^2^ and 46.13 Å^2^ compared to Wat1 B-factors 34.26 Å^2^ and 39.77 Å^2^ in chains A and B, respectively) indicative of weaker binding. In the remaining molecule (chain B of dataset 2) this water molecule is absent and the Zn1 site is clearly tetrahedral. The Zn2 site also differs between the four molecules. In the lower resolution structure (dataset 1) this is clearly tetrahedral (four co-ordinate) in molecule A, while in molecule B a second, “apical” water molecule (Fig 6A, Wat3) is present, as observed in other MBL structures [43, 46, 55], to create a pentaco-ordinate center. In the higher resolution structure solved from dataset 2 the position of Wat3 is occupied by chloride ions in both chains, and in both cases Zn2 is five co-ordinated (Fig 6B).

**Fig 7:**
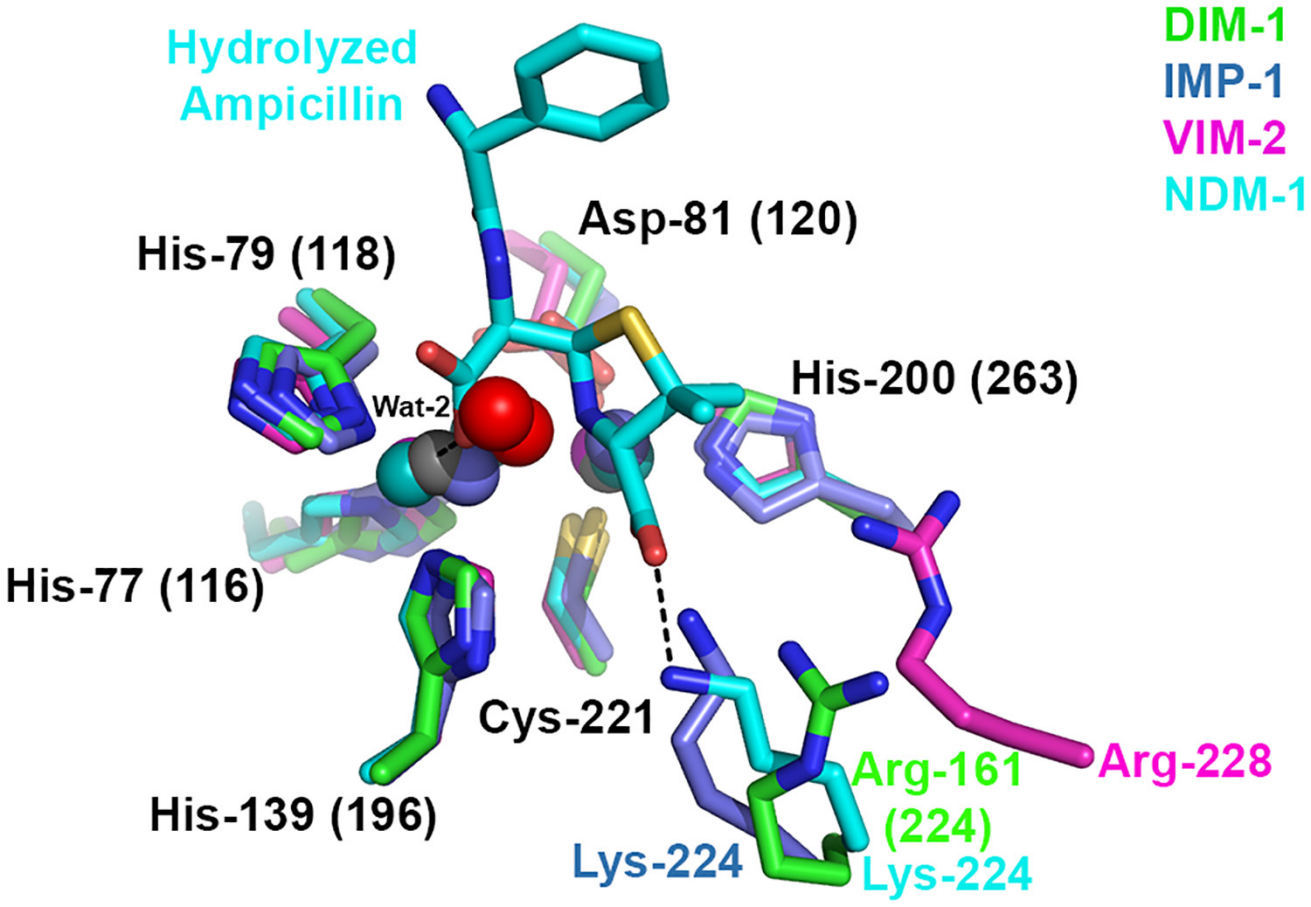
Overlay of B1 Metallo-β-Lactamase Active Sites Showing Hydrolyzed β-Lactam Binding. Figure shows DIM-1, IMP-1 (1DDK [23], carbon, zinc and waters teal); VIM-2 (1KO3 [44], carbon, zinc and waters magenta) and NDM-1:ampicillin complex (3Q6X [41], carbon, zinc and waters cyan). Interactions of hydrolyzed ampicillin with NDM-1 are shown as dashed lines. Note differing positions of Arg-161 (224) (DIM-1), Lys-224 (IMP-1, NDM-1) and Arg-228 (VIM-2). This Figure was generated using Pymol.

Five co-ordinate Zn1 sites have been observed in some other B1 MBL crystal structures, notably those of IND-7 (Fig 6C, [47]) and reduced VIM-2 (Fig 6D, [44]). Notably, Yamaguchi *et al* [47] used comparisons with structures of biomimetic copper complexes [56] to describe the co-ordination geometry about IND-7 Zn1. Calculation of the structural parameter τ ((β − α)/60), where angle α refers to His79 (118) ND1—Zn1—His139 (196) NE2 and β to O (Wat2)—Zn1—His77 (116) NE2, can be used to represent the distortion from square to trigonal pyramidal co-ordination geometry. (The value of τ is considered to be 0 for an ideal square pyramid and 1 for an ideal trigonal bipyramid). On this basis the IND-7, reduced VIM-2 and DIM-1 Zn1 sites can all be described as distorted trigonal bipyramidal, with values of τ all above 0.85. In all three structures Wat2 is relatively distant from Zn1 compared to other ligands, consistent with proposals [47] that this defines the position occupied by the carbonyl oxygen of incoming β-lactams and hence may be readily displaced when substrates bind. Superposition with structures of NDM-1 complexes with hydrolysed β-lactams (e.g. 3Q6X; [41] Fig 7) indeed locates DIM-1 Wat-2 in the approximate position of the C7 carboxylate formed on β-lactam hydrolysis and thus supports this assertion.

The structural parameter τ can also be used to describe the geometry of the Zn2 site. As described above, defining angles α as (Wat1 (Cl^-^))—Zn2—His200 (263) NE2 and β as O (Wat3 (Cl^-^))—Zn2—Asp81 (120) OD2 gives values of τ ranging from 0.18 to 0.37 for the three chains where Zn2 is five-co-ordinate. This indicates that the DIM-1 Zn2 site can be described as a distorted square pyramid rather than the trigonal bipyramid observed in e.g. VIM-2 (Fig 6D pdb 1KO3 [44]). Under these criteria the Zn2 sites of NDM-1 (Fig 6E, pdb 3SPU [54]) and CcrA (pdb 1ZNB [46]) can also be described as square pyramidal. An additional contrast is provided by the IND-7 (Fig 6C [47]) and GIM-1 [48] structures, where in both cases Zn2 is tetrahedrally co-ordinated, as observed in one of the four DIM-1 chains described here.

The separation of the two active site metal ions in the four DIM-1 structures ranges from 3.97 Å (dataset 1 chain A) to 4.39 Å (dataset 2 chain B). While comparison with other structures is complicated by the lack of atomic resolution data (overall coordinate errors based on R-values are 0.36 Å, dataset 1, and 0.13 Å, dataset 2), these values appear to be at the upper end of the range observed in other B1 MBL structures, which for uncomplexed enzymes lies more typically in the 3.5–3.7 Å range (e.g. IND-7 3.64 Å, pdb 3L6N [47]; GIM-1 3.45–3.50 Å pdbs 2YNT and 2YNW [48]) and see recent near-atomic resolution structures of BcII (3.50 Å, 1.20 Å resolution, pdb id 4C09) and VIM-2 (3.51 Å, 1.29 Å resolution, pdb id 4BZ3). Increased separations have been observed for structures determined under more acidic conditions [16, 57], and the metal—metal distance is also observed to increase in some complexes with hydrolyzed β-lactams (e.g. NDM-1:ampicillin pdb 3Q6X 4.58 Å [41] compared to unliganded NDM-1 3.56–3.97 Å pdb 3SPU), indicating that the MBL active site architecture is able to adapt to accommodate bound ligands. It is notable that other structures in which, as is the case for dataset 2, chloride ions are observed in the MBL active site (e.g. 1KO3 [44] or 2BG2 [57]) also feature relatively large separations between the two zinc ions (4.20 Å and 4.09 Å respectively). Our observation of a variety of metal co-ordination arrangements in DIM-1, and the range of architectures observed in the zinc sites of other enzymes, together highlight the flexibility of co-ordination geometry about both metal ions of the B1 MBL active site (for a review see [58]). This is also consistent with proposals that metal co-ordination geometry can change during β-lactam binding and hydrolysis [59–61].

In common with some other mobile B1 MBLs (e.g. GIM-1 and TMB-1; [7, 13]) DIM-1 features an arginine at residue 161 (224), rather than the lysine found at the equivalent position in most other enzymes (e.g. NDM-1, IMP-1, SPM-1, IND-7). Structures of inhibitor and β-lactam complexes of the IMP-1 and NDM-1 enzymes [17, 23, 41], as well as evidence from directed mutagenesis studies [62, 63], implicate this lysine in substrate interactions via the invariant carboxylate at the β-lactam C3 (penicillin) or C4 (cephalosporin) position. Structural comparisons (Fig 7) suggest that DIM-1 Arg161 (224) NH1 might make similar contacts, although we note that, relative to Zn2 (also implicated in binding substrate carboxylate) DIM-1 Arg161 (224) NH1 is not identically located to Lys224 NZ of e.g. IMP-1 or NDM-1. Steady-state kinetic data [7, 10, 13] show enzymes with Arg224 to be less efficient than other B1 MBLs against some substrates, in particular penicillins (for which *k*_*cat*_ values are notably reduced [7]). It is possible that interactions involving Arg224, rather than Lys224, result in binding of some substrates in orientations that are less optimal for hydrolysis.

In summary, the DIM-1 crystal structure presented here displays similarities and differences when compared to those for other B1 MBLs. While the overall fold and binuclear zinc center are common to all B1 MBLs, the DIM-1 structure highlights the variability evident in the active site architecture around the L3 and L10 loops and in the identity and location of residues (DIM-1 Arg161 (224)) likely to be involved in binding the β-lactam C2/C3 carboxylate. Moreover, our data provide further evidence of variability in the co-ordination of both of the catalytic zinc ions in B1 MBLs. Possession of a relatively plastic active site which differs significantly between individual enzymes is one reason why MBLs remain challenging targets for inhibitor design. The availability of structural information for enzymes, such as DIM-1, whose active sites contain such distinguishing features and that, by virtue of their mobilization and presence in pathogens, are a present challenge to clinical β-lactam use, is thus a stepping stone to development of broad spectrum inhibitors effective across the full range of MBLs.
